# 

**Published:** 1917-07-28

**Authors:** 


### CONTENTS.

| | PAGE |
|---|---|
| **The B.M.A. Annual Representative Meeting** | 329 |
| **The Responsibility of the Anæsthetist** ... | 330 |
| **Hospital and Institutional News** ... ... ... | 331 |
| The Prince of Wales' Fund: Reform it, or end it—Another Light on Mesopotamia—A Soldier Patient's Invention—Dr. Keats' Pension—The London Hospital and Air-Raids—Attempt to Bribe a Hall Porter—A Physician Benefits his Hospital—Aid for Women Medical Students—The Food Required by the Nation—Liverpool's Hospital Saturday Fund—A Progressive Institution—The Personal Service Tradition—Holidays and Hospital Patients—Private Wards in Cottage Hospitals—Hospital Water Supply—Twenty-nine Years' Service—Poor-Law Officers and a Ministry of Health—Guy's Dental Surgeons and Military Service—The Need for More Sanatorium Beds—The Infant Death-rate at Warrington—Health in Jeopardy—Where Economy Fails—The Health of the Children of Sutton Coldfield—The Dearth of Dentists—Rats and the Port of Liverpool—Fumigation Recommended—This Week's Drug Market—To Our Readers. | |
| **The Worst War of the World** ... ... ... | 337 |
| Its Teachings and Momentous Issues. | |
| **The War Cripple** ... ... ... ... ... | 338 |
| His Restoration, Occupations, and Future. | |
| **Subacute Endocarditis in Children** ... ... | 339 |
| **Lessons in Public Morals** ... ... ... ... | 341 |
| Three Plays with a Purpose. | |
| **The Work of Hospital Almoners—III.** ... ... | 342 |
| Address by Miss Cummins at the London School of Economics. | |
| **The Matrons' and Sisters' Department** ... | 343 |
| The College of British Nursing. | |
| Round the Hospitals. | |
| A Hospital Food Supervisor—Nursing Sisters of St. John the Divine—District Nurses in War-time—Work for the Educated Woman—The "Choker" College in Red Cross Nursing. | |
| **National Health Work for Women** ... ... | 346 |
| **Leicester Royal Infirmary** ... ... ... ... | 346 |
| **Parliament and the Profession** ... ... ... | 348 |
| **Passing Events and Topics** ... ... ... ... | 348 |
| **Tuberculosis Annual Reports** ... ... ... | iv |
| **Homes for Rescued Children** ... ... ... | iv |
| **Matrons' and Sisters' Appointments** ... ... | iv |
| **The Book Market** ... ... ... ... ... | iv |
| **Memorial to Dr. Potter** ... ... ... ... | iv |
| **The Institutional Worker** ... ... (Suppl.) | 1 |
| The Probationer's Training and Time-Table: Answer to May Question. Question for July. | |

